# Incidental Detection of Bowen’s Disease in a Patient Presenting With Dermatophytosis: A Case Report of Two Distinct Back Lesions

**DOI:** 10.7759/cureus.96810

**Published:** 2025-11-13

**Authors:** Balachandra S Ankad, Balkrishna P Nikam, Sourab Dhananjaya

**Affiliations:** 1 Dermatology, S Nijalingappa Medical College, Bagalkot, IND; 2 Dermatology, Krishna Institute of Medical Sciences (Deemed to Be University), Karad, IND; 3 Dermatology, Subbaiah Institute of Medical Sciences and Research Centre, Shivamogga, IND

**Keywords:** bowen’s disease, dermatophytosis, dermoscopy, incidental diagnosis, squamous cell carcinoma in situ

## Abstract

Bowen’s disease (squamous cell carcinoma in situ) is an intraepidermal malignancy that can clinically resemble benign inflammatory or infectious dermatoses. The coexistence of dermatophytosis and Bowen's disease can make diagnosis challenging. A 60-year-old man presented with itchy, scaly lesions on the upper back, clinically consistent with tinea corporis. Examination also revealed a separate dark plaque with well-defined borders, distinct from the dermatophytic lesions. Dermoscopy showed eccentric glomerular vessels with brown pigmentation, raising suspicion for Bowen's disease. The fungal infection was treated with standard antifungal therapy, and the Bowen’s lesion was managed with topical 5-fluorouracil (5-FU). This case highlights the importance of thorough skin examination, and dermoscopic assessment proved particularly useful. Bowen’s disease may occur without noticeable symptoms, and dermoscopy can help distinguish benign from malignant lesions by revealing characteristic features, making it a valuable tool for improved patient management.

## Introduction

Bowen's disease, a squamous cell carcinoma in situ, represents full-thickness epidermal atypia with an intact basement membrane. It typically occurs on sun-exposed areas of older adults, although it may develop anywhere on the skin, including less-exposed sites such as the trunk and back [1]. Risk factors include chronic ultraviolet exposure, arsenic exposure, immunosuppression, human papillomavirus infection, and chronic irritation [2]. Clinically, it often presents as a slowly growing erythematous or pigmented scaly plaque and is known for mimicking benign dermatoses such as psoriasis, eczema, or chronic dermatophytosis. Because of its indolent course, it may remain unnoticed or misinterpreted for years.

We report an interesting case in which a Bowen's disease plaque was incidentally detected during the evaluation of superficial dermatophytosis on the back. This case highlights the importance of thorough skin examination and the use of dermoscopy to identify concomitant lesions.

## Case presentation

**Figure 1:**
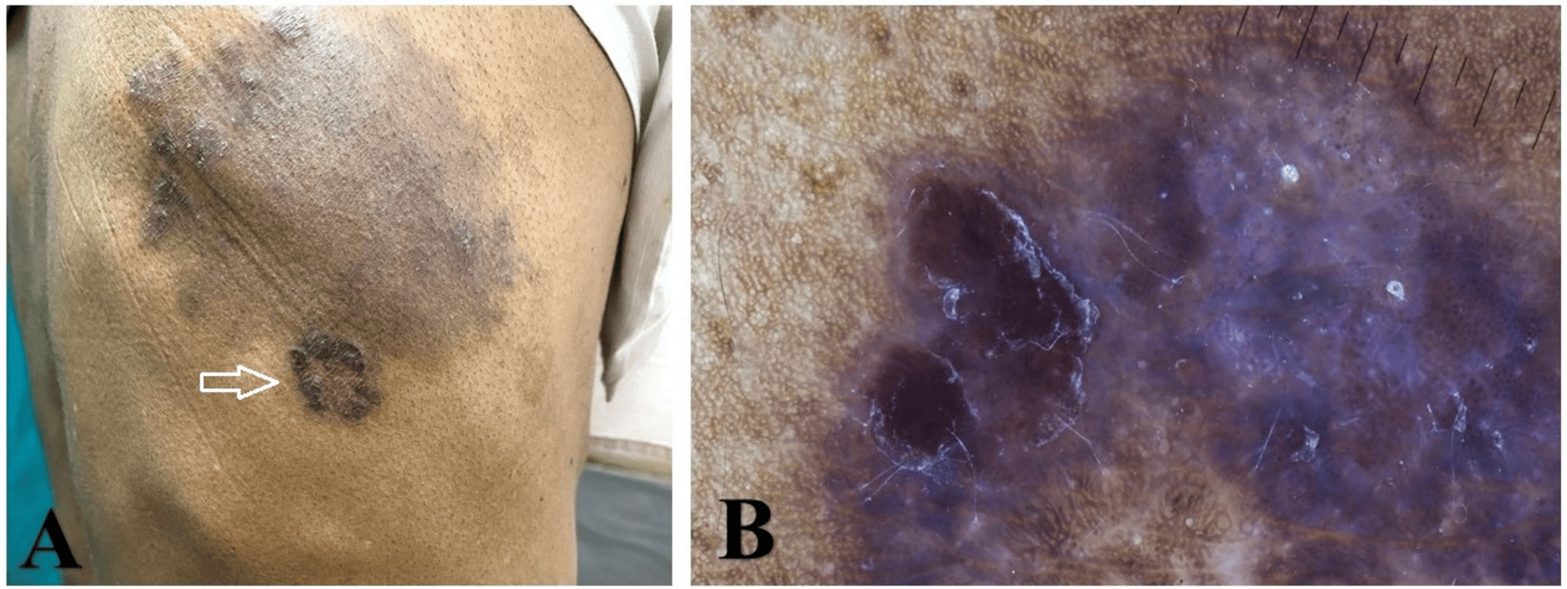
Clinical and dermoscopy images (A) Clinical image of the patient with Bowen's disease and dermatophytosis (arrow). (B) Dermoscopy of Bowen's disease showed eccentric, clustered glomerular vessels, dark brown areas, a bluish hue, brown dots in the periphery, and white scales.

**Figure 2:**
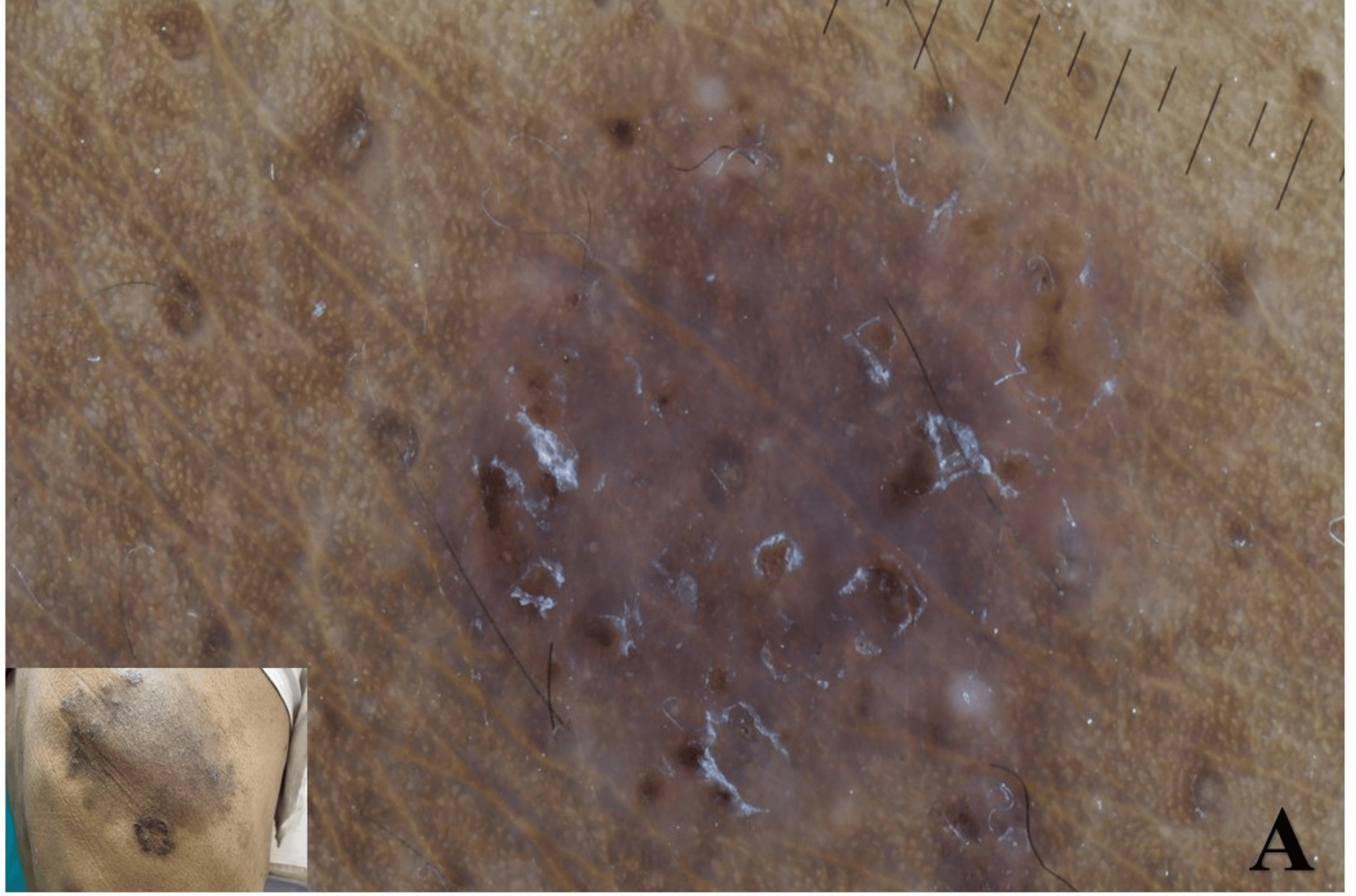
Dermoscopy image of the dermatophytosis (A) Dermoscopy of the dermatophytosis showed brown globules with periglobular and perifollicular scaling. The scales were arranged in semicircular or circular patterns. The inlet shows the clinical image and the site of dermoscopy, with the circle indicating the exact area examined.

A 60-year-old man presented to the dermatology outpatient department with complaints of itch and scaling over the lesions on the upper back for the last three months. No history of similar lesions elsewhere, history of radiation or chronic arsenic ingestion, or any immunosuppression was present. There was no history of systemic symptoms such as fever, weight loss, or malaise. Upon clinical examination, there was an annular erythematous scaly plaque with active margins and central clearing over the upper back, consistent with tinea corporis. Adjacent to the tinea lesion, a well-defined dark brown plaque measuring approximately 3 × 2 cm was incidentally seen over the right upper back. The plaque was asymptomatic, was mildly elevated with rough scaling, and had an irregular but well-defined periphery (Figure 1A). This lesion was not known to the patient, who presumed all the scaling patches belonged to the same fungal infection. No regional lymphadenopathy was noted. Polarized dermoscopy of the dark plaque demonstrated eccentric, clustered glomerular vessels, dark brown areas, a bluish hue, brown dots in the periphery, and white scales, typical features of Bowen’s disease (squamous cell carcinoma in situ) (Figure 1B). Dermoscopy of the surrounding annular lesions revealed scattered brown clods/globules and periglobular, perifollicular white scales on the brownish background, which are typical features of tinea corporis (Figure 2A).

**Figure 3:**
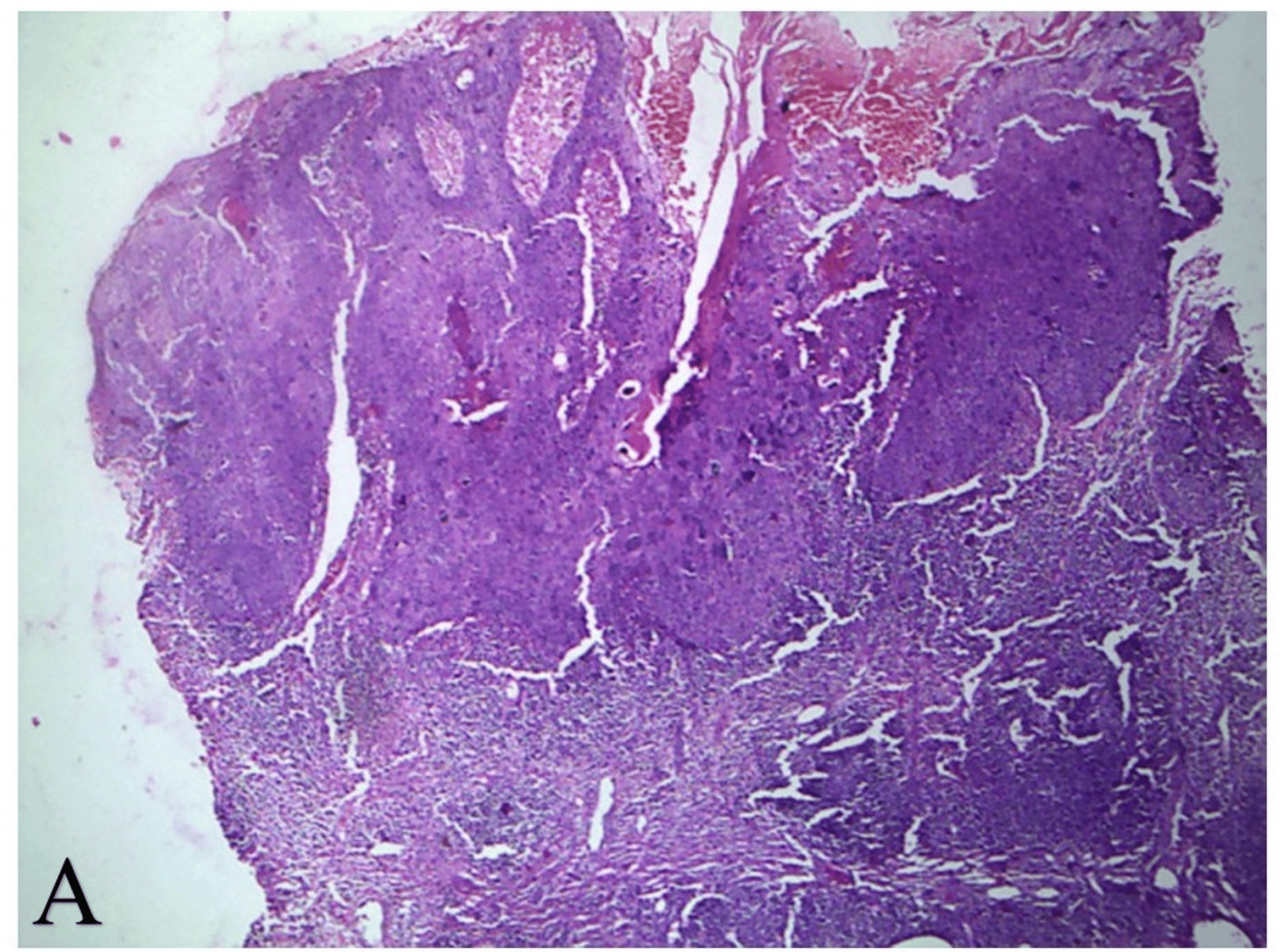
Histopathology of Bowen's disease (A) Histopathology of Bowen's disease showed a "windblown" appearance with complete replacement of keratinocytes in the epidermis, and marked atypia is well appreciated (H&E, 10×).

**Figure 4:**
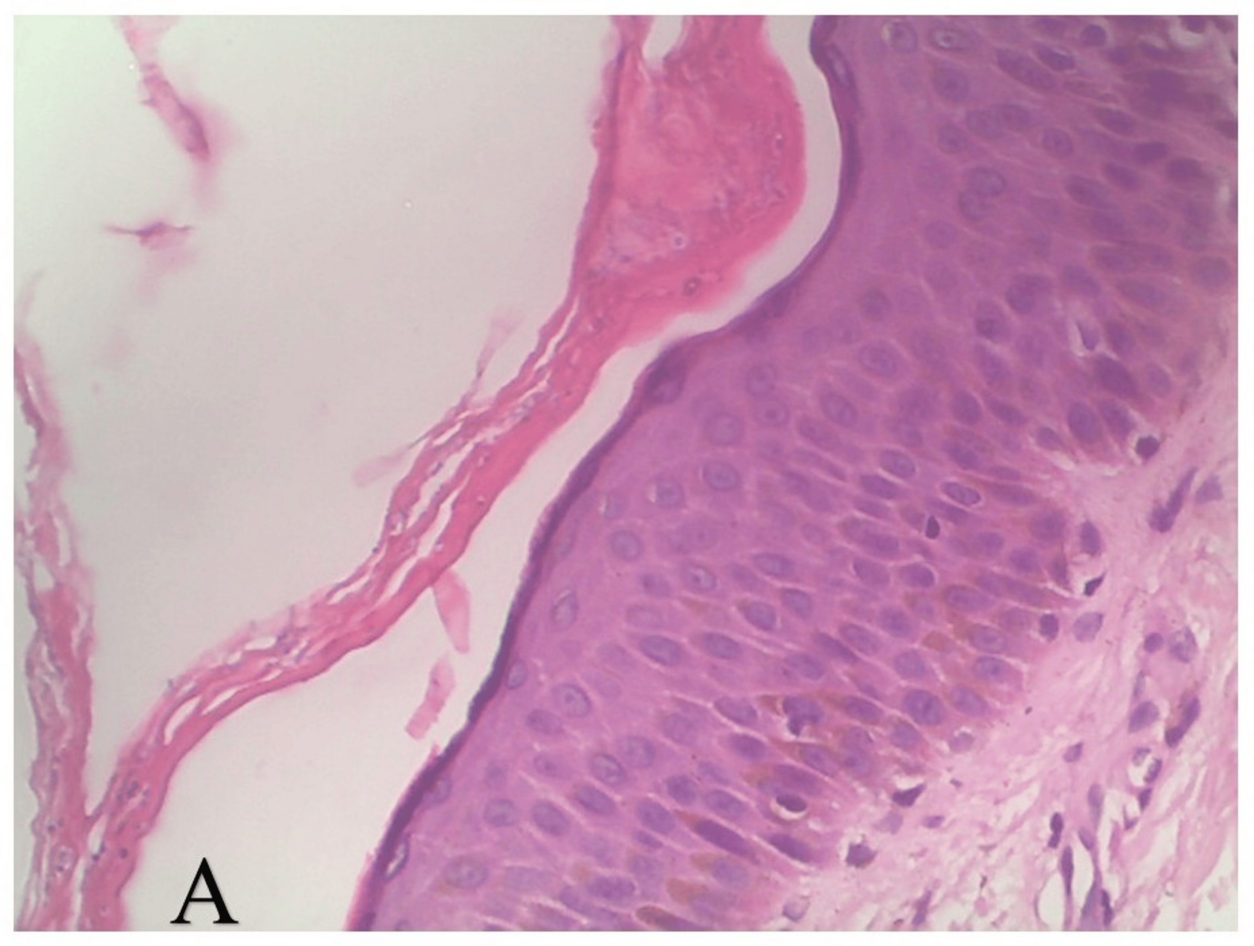
Histopathology of dermatophytosis (A) Histopathology of the tinea corporis demonstrated fungal hyphae in between the layers of the stratum corneum (H&E, 40×).

A KOH mount prepared from the scaly margins of the annular plaques was positive for septate branching hyphae, hence confirming the diagnosis of dermatophytosis. Biopsy from the smaller plaque revealed features of Bowen’s disease with a "windblown" appearance and complete replacement of epidermal keratinocytes by atypical cells (H&E, 10×) (Figure 3A). In contrast, biopsy from the larger plaque showed features of dermatophytosis with fungal hyphae seen between the layers of the stratum corneum (H&E, 40×) (Figure 4A).

We had prescribed oral supra bioavailable itraconazole 65 mg capsule once daily for four weeks, along with topical luliconazole 1% cream once daily and oral levocetirizine 5 mg once daily for the management of dermatophytosis. Regarding Bowen’s disease, we advised the application of topical 5-fluorouracil (5-FU) 5% cream once nightly for six weeks.

## Discussion

Bowen's disease is an intraepidermal carcinoma with slow progression and a 3%-% risk of transforming into invasive squamous cell carcinoma if left untreated [1,2]. It may appear on any part of the body, including the back, a site that is often inadequately examined during routine skin checks, leading to delayed diagnosis until symptoms arise. In this case, the patient was admitted for evaluation of dermatophytosis, and the Bowen's lesion was incidentally found during clinical examination. This emphasizes the importance of a full skin examination and the value of dermoscopy in identifying incidental or concomitant lesions in everyday practice.

The coexistence of dermatophytosis and Bowen's disease is uncommon and can be challenging to diagnose, as both may present with erythema, scaling, and irregular margins. Although the lesions were spatially distinct, they occurred concurrently, and the patient had assumed they were part of a single spreading fungal infection. This overlap demonstrates how symptom-focused consultations may obscure silent malignancies and delay diagnosis. A dermoscopic hallmark of Bowen's disease, clustered glomerular vessels on a brown background, was a key feature that distinguished it from fungal or inflammatory dermatoses, where such vascular patterns are rarely observed [3-5].

Bowen’s disease may be treated with excision, cryotherapy, photodynamic therapy, imiquimod, or topical 5-FU. For small, well-defined lesions in cosmetically sensitive or challenging areas, topical 5-FU is a non-invasive and effective option, with reported response rates of 80%-90% and low recurrence [6,7].

Dermatophytosis shows typical dermoscopic patterns, such as brown globules with periglobular and perifollicular scales, arranged in semicircular or circular patterns [8]. Dermoscopy therefore provided helpful support in confirming the clinical suspicion.

This case reinforces several important dermatologic principles. A complete skin examination should be performed for every patient, regardless of the primary complaint, as incidentally detected lesions may carry significant diagnostic or prognostic value. Dermoscopy remains a valuable non-invasive tool for distinguishing neoplastic lesions from infectious or inflammatory dermatoses, aiding earlier and more accurate diagnosis. Ultimately, this case highlights the need for clinical vigilance and the reminder that not all scaly or itchy plaques are fungal in origin.

## Conclusions

In this case, we report the incidental detection of Bowen's disease in a patient admitted for dermatophytosis of the back. Two separate lesions coexisted but were distinct in nature, which initially caused diagnostic confusion. This case highlights that not every itchy, scaly plaque is fungal in origin, and that clinical vigilance, together with dermoscopic evaluation of all lesions, including incidental ones, is essential for the early detection of skin neoplasms.
